# Mood instability, depression, and anxiety in pregnancy and adverse neonatal outcomes

**DOI:** 10.1186/s12884-021-04021-y

**Published:** 2021-08-25

**Authors:** Hua Li, Angela Bowen, Rudy Bowen, Nazeem Muhajarine, Lloyd Balbuena

**Affiliations:** 1 College of Nursing, University of Saskatchewan, Health Sciences Building, E-Wing, Room 4248, 104 Clinic Place, SK S7N 2Z4 Saskatoon, Canada; 2 Department of Psychiatry, University of Saskatchewan, Ellis Hall, RUH, Room 112, SK S7N 0W8 Saskatoon, Canada; 3 Department of Community Health and Epidemiology, University of Saskatchewan, Health Sciences Building, E-Wing, Room 3246 104 Clinic Place, SK S7N 2Z4 Saskatoon, Canada; 4 Department of Psychiatry, University of Saskatchewan, Ellis Hall, RUH, Room 104, SK S7N 0W8 Saskatoon, Canada

**Keywords:** Antenatal women, Mood instability, Depression, Anxiety, Neonatal outcomes

## Abstract

**Background:**

Antenatal women experience an increased level of mood and anxiety symptoms, which have negative effects on mothers’ mental and physical health as well as the health of their newborns. The relation of maternal depression and anxiety in pregnancy with neonate outcomes is well-studied with inconsistent findings. However, the association between antenatal mood instability (MI) and neonatal outcomes has not been investigated even though antenatal women experience an elevated level of MI. We sought to address this gap and to contribute to the literature about pregnancy neonate outcomes by examining the relationship among antenatal MI, depression, and anxiety and neonatal outcomes.

**Methods:**

A prospective cohort of women (*n* = 555) participated in this study at early pregnancy (T1, 17.4 ± 4.9 weeks) and late pregnancy (T2, 30.6 ± 2.7 weeks). The Edinburgh Postnatal Depression Scale (EPDS) was used to assess antenatal depressive symptoms, anxiety was measured by the EPDS anxiety subscale, and mood instability was measured by a visual analogue scale with five questions. These mood states together with stress, social support, as well as lifestyle were also examined in relation to neonatal outcomes using chi-square tests and logistic regression models.

**Results:**

Mood instability, depression, and anxiety were unrelated to adverse neonatal outcomes. Only primiparous status was associated with small for gestational age after Bonferroni correction.

**Conclusions:**

We report no associations between antenatal mood symptoms including MI, depression, and anxiety and neonatal outcomes. More studies are required to further explore the relationship between antenatal mood instability, depression, and anxiety and neonatal outcomes.

**Supplementary Information:**

The online version contains supplementary material available at 10.1186/s12884-021-04021-y.

## Background

Pregnancy and childbirth are often viewed as joyful events, but they can also be overwhelming and challenging for some mothers. Becoming a mother is a vulnerable period for women to develop perinatal depression, anxiety [1, 2], postpartum blues, baby pinks [3], and mood instability (MI) [4, 5]. Perinatal women often experience the highest euphoric, irritable, and depressed moods in early pregnancy and again around the time of giving birth [6, 7]. This phenomenon is thought to be triggered by the large hormonal fluctuations occurring at these times [6, 8, 9].

Ample studies have found that not only do maternal depression and anxiety adversely affect a mother’s physical and mental health, but also the infant’s physical and cognitive development, and the mother-infant relationship, all of which endure into childhood and beyond [10]. The impact of antenatal depression or anxiety on neonatal outcomes has been investigated extensively in both developing and developed countries. However, the findings are inconsistent. Some studies have found a significant relationship between antenatal depression or anxiety and low birth weight (LBW), preterm birth (PTB), small for gestational age (SGA), or low Apgar score [11–14], while others have not [15–18]. These inconsistent findings could result from differences in methodological factors including measurements, the timing of assessments, composition of samples, and other confounders [19, 20].

In addition to depression and anxiety, mood instability has been found to be a prominent feature of pregnant and postpartum women. For example, in a longitudinal study, Bowen, A. et al. [21] investigated MI in a group of pregnant women into early postpartum compared to a control group of non-pregnant women with normal menstruum at 16 weeks, and 32 weeks of pregnancy, and 4 weeks of postpartum. The study found that perinatal women were more likely to experience MI (depressed, irritable, anxious, and euphoric moods). The relationship between MI and fluctuation of ovarian hormones has been studied largely under the concept of postpartum blues (PPB). The etiology of PPB is still undetermined although a biological underpinning – the rapid decline in ovarian hormones following delivery - has been proposed [22]. However, the correlation of PPB with ovarian hormones is neither consistent nor well-studied [23]. There is a lack of research regarding the relationship between perinatal MI and ovarian hormonal fluctuation.

Mood instability, defined as “rapid oscillations of intense affect, with a difficulty in regulating these oscillations” [24], has a prevalence of 13.9% among UK adults according to the Psychiatric Morbidity Survey [25]. The term MI is often used interchangeably with mood swings, affect instability, and emotional dysregulation [24, 26]. With MI affecting a significant part of the population, its relation with both psychological and psychopathological traits has been examined including negative affect [27], low self-esteem [28], neuroticism [29]. There is growing recognition that MI increases the risk for an array of psychiatric disorders including depression and anxiety [30–32].

A recent study reported cross-sectional associations between MI and depression in early and late pregnancy, and MI in pregnancy predicted postpartum depression independently of antenatal depression [33]. In addition, Hapgood et al. [34] found that emotional lability observed in the early postpartum was a strong predictor of depression up to 14 months postpartum (13 weeks, 26 weeks, and 60 weeks). Although neonatal outcomes and antenatal depression and anxiety have been well-studied, we are not aware of any study that investigates the relationship between MI in pregnancy and neonatal outcomes.

In this study, we addressed the research gap and contribute to the literature about pregnancy neonate outcomes by examining the relationship among antenatal MI, depression, and anxiety and neonatal outcomes.

## Methods

### Participants and procedure

The current study was a secondary data analysis of the Feelings in Pregnancy and Motherhood Study (FIP) [33]. Maternal information was collected in early pregnancy (T1: 17.4 ± 4.9 weeks) and late pregnancy (T2: 30.6 ± 2.7 weeks) while information of neonatal outcomes was obtained from the mothers and linked with hospital discharge records. The present study analyzed data from 555 mother-baby dyads. More information on the FIP can be found in a previous publication [33].

The Behavioural Research Ethics Board at the University of Saskatchewan has approved this study. All participants signed an informed consent form.

### Measures

We assessed the symptoms of three constructs known in the literature to be closely related: MI, depression, and anxiety, making use of thresholds for clinical relevance.

#### Depression

Depressive symptoms were measured using the Edinburgh Postnatal Depression Scale (EPDS) at T1 and T2 [35]. The EPDS is the most widely used self-report measure for screening depression among pregnant and postpartum women [36, 37]. Respondents select one of four possible responses (0-3) to each of ten questions to indicate how they felt in the previous week. Ratings for each item are summed for a possible maximum score of 30 (range 0-30; 0 = not depressed, 30 = highest score for depressive mood). Validation studies of the EPDS among antenatal women have shown a high degree of internal consistency (Cronbach’s α = 0.914), and good convergent validity with the Beck Depression Inventory-I (rho = 0.850, *p* < 0.001) [38], while EPDS has been found to have a sensitivity of 77% and specificity of 94% [38]. In this study, attaining a score ≥ 12 at either T1 or T2 was indicative of clinically significant depression [39].

#### Anxiety

Anxiety symptoms were measured at T1, and T2 using the EPDS anxiety subscale (items 3, 4, and 5) [40–43] with a score range of 0 to 9. Among antenatal women, studies found that the anxiety subscale has an acceptable internal consistency (Cronbach’s α = .77) [44], and (Cronbach’s α = .74) respectively. In this study, we followed the recommended cut-off score of ≥ 6 for community samples [45] as indicative of clinically significant anxiety.

#### Mood instability

Mood instability was measured using five questions that were answered on a Visual Analogue Scale (VAS): 1 “mood frequent ups and downs”, 2 “mood swings occur for no reason”, 3 “other people complain about your mood swings”, 4 “having trouble following through with plans because of mood swings”, and 5 “not making commitments because moods might change” [33]. Each question is awarded a score ranging from 0 to 10, and the higher score indicates a higher level of MI symptoms with a possible total score of 50. We dichotomized MI scores into above and below the mean. This was done by calculating the average of T1 and T2 scores separately. If a woman’s score was above the mean in either period, her MI symptom was coded 1 “High MI” and 0 “Low MI” otherwise.

#### Other maternal variables

Demographic, psychosocial, and pregnancy-related variables were also assessed. These included age (< 25 vs ≥ 25 years), education attainment (< Grade 12 vs ≥ Grade 12), marital status (with or without a partner), ethnicity (aboriginal or not aboriginal), and annual family income (≤ 40K vs > 40K). The psychosocial variables were sources of social support (1 or less vs 2 or more), the number of stressors (0-2 vs > 2), partner support (i.e., is your partner supportive? yes or no), maternal smoking (yes or no), alcohol consumption (yes or no), and physical activity status (yes or no) during pregnancy were assessed. As we did with the mood variables, we classified a woman as having a risk factor if these were reported at either T1 or T2. We also used parity (primiparous vs multigravida) as a predictor variable.

#### Neonatal outcome variables

Neonatal outcome variables included one- and five-minute Apgar scores, low birth weight (LBW), small for gestational age (SGA), and preterm birth (PTB). We dichotomized the Apgar scores based on previous studies [46, 47]. Apgar scores reflect a baby’s wellbeing at birth by assessing five areas: activity, pulse, grimace, appearance, and respiration [48]. Each area is rated from 0 to 2 with a possible score of 10 (scores 7 or above indicate that the baby is doing well). Apgar scores were dichotomized into below normal < 7 or normal ≥ 7 [48]. Birth weight was measured in grams (g) and dichotomized into two levels: low < 2500 g or normal ≥ 2500 g [49]. SGA was defined as the infant with birth weight below the 10^th^ percentile of mean body weight of the infants of same gestational age and same gender [50]. Preterm birth is based on the duration of pregnancy in completed weeks from the first day of the last normal menstrual period to birth. The variable was dichotomized into preterm birth (PTB) (< 37 weeks) and normal term birth (≥ 37 weeks) [51].

### Data analysis

We cross-tabulated nine maternal variables in pregnancy with the five neonatal outcomes. Chi-square or Fisher’s exact test was used to examine the association of each maternal variable and each neonatal outcome (45 tests in all). To protect against false positive results from a large number of tests, we set α at .001 for each test (i.e., family-wise α = .05). For each result that was significant at 0.001, we created a logistic regression model that adjusted for maternal age category and marital status. Each logistic regression model was performed first, with complete cases and then with multiple imputations. Coefficients in logistic regression models were exponentiated to yield odds ratios (OR), and their 95% confidence intervals (CI) were calculated accordingly. Multiple imputation was necessary because some variables (SGA, Apgar 1 minute, Apgar 5 minute, LBW, pregnancy stress, and maternal age category) had missing values that ranged from less than 1 to 17 percent. Multiple imputation is a principled way of avoiding biased estimates that can result from non-random missingness and overly narrow confidence intervals [52]. For sensitivity analysis, logistic regression was repeated with modification that the independent variables (depression, anxiety, and MI) were treated as continuous variables.

Statistical analyses were performed using Stata and imputation was carried out using multiple imputation with chained equations (MICE) [53]. We created 20 imputed datasets following the recommendation of White and colleagues [54].

## Results

### Characteristics of the mothers and babies

A total of 555 women participated in this study with a mean age of 29.0 years (*SD* = 4.85), and 38% (n = 212) were first-time mothers. Most of the women lived with a partner, had a Grade 12 or higher education, and had a family income of at least $40,000. Sixty-eight babies (12%) had a 1-minute Apgar score < 7, while 15 (3%) babies had a 5-minute Apgar of <7. Only 20 (4%) of the babies’ birth weight was < 2500g, while 39 babies (7%) were SGA. Thirty-two babies were born preterm (6%) (Table 1).

**Table 1:** Characteristics of mothers and infants (N=555)

Mothers	Number	Percent
***Demographic variables***		
Age (≥ 25)	465	84
Living with a partner	504	91
Aboriginal ancestry	42	8
Grade 12 or above	529	95
Family income > $40,000	263	90
***Mood symptoms***		
Clinically significant depression	109	20
Clinically significant anxiety	118	21
Above-average mood instability	283	51
***Psychosocial and behaviour variables***		
Engaged in regular physical activity	379	32
Alcohol use	57	10
Cigarettes smoking	72	13
With partner’s support	516	93
Stress	304	55
First-time mothers	212	38
**Infants**		
***Neonatal outcome variables***		
Apgar 1 minute score <7 ^a^	68	12
Apgar 5 minute score <7 ^a^	15	3
Pre-term birth (< 37 weeks)^b^	32	6
Low birth weight (<2.5 kg) ^b^	20	4
Small for gestational age^b^	39	7

^a^Had 17% missing values

^b^Had 10% missing values

One hundred and nine women (20%) were screened positive for depression according to the cut-off 12 or more on EPDS, 118 (21%) experienced high anxiety (EPDS anxiety subscale score ≥ 6), and 283 (51%) had an above average MI score. Approximately 1/3 of women exercised regularly, while over 90% of women received partner support. Over half of women (n = 304) experienced stress, while 10% of (n = 57) and 13% of (n = 72) used alcohol and smoked cigarettes respectively (Table 1).

The results of Cronbach’s α for MI questionnaire are 0.886 at T1 and 0.837 at T2, for EPDS are 0.843 at T1 and 0.781 at T2, and for EPDS anxiety subscale are 0.726 at T1 and 0.702 at T2, indicating good or acceptable internal consistency for the measures.

### Mood instability, depression, anxiety, other maternal factors, and neonatal outcomes

For the univariate logistic regression, anxiety in pregnancy was associated with Apgar (5-minute) scores below 7 (χ^2^=3.78, p = .05), while stress was associated with low Apgar scores (1-minute) (χ^2^=5.09, p = .02). Smoking, lack of partner support, and primiparous status were each associated with SGA (χ^2^ =10.05, *p* = .002; χ^2^ = 7.63, *p* = .006; χ^2^ = 13.25, *p* < .001 respectively). In addition, primiparous status was associated with LBW (χ^2^ = 4.50, *p* = .03). Mood instability, depression, alcohol use, and exercise during pregnancy were not associated with any of the neonatal outcomes, while PTB was not associated with any maternal variables. The complete crosstabulation of maternal variables and neonate outcomes is presented in Table 2.

**Table 2:** Cross-tabulation of adverse neonatal outcomes by maternal variables (entries are percentages (%))

Variables	Measure	Apgar 1 minute score <7 ^a^	Apgar 5 minute score <7 ^a^	Pre-term birth (< 37 weeks)^b^	Low birth weight (<2.5 kg) ^b^	Small for gestational age^**b**^
Depression	EPDS < 12	14.78	3.23	6.39	4.36	8.11
	EPDS ≥ 12	14.61	3.37	6.45	2.13	6.52
Anxiety	EPDS Anxiety subscale < 6	13.86	2.45	6.03	3.72	7.79
	EPDS Anxiety subscale ≥ 6	18.28	6.45	7.84	4.81	7.92
Mood instability	≤ average	14.03	3.62	6.02	4.00	8.84
	> average	15.48	2.93	6.80	3.91	6.83
Alcohol use	Not at all	14.22	3.61	6.21	4.16	7.98
	Yes, at some point	20.00	0.00	8.33	2.04	6.38
Smoking	No	15.11	3.36	6.05	3.76	6.50
	Yes	11.36	2.27	9.26	5.45	18.87
Stress	0-2 stressors	10.75	3.29	6.99	4.29	9.57
	3+ stressors	18.22	3.23	5.90	3.65	6.32
Partner support	Yes	14.48	3.22	5.94	3.77	7.01
	No	19.23	3.85	13.79	6.90	21.43
Parity (First-time mothers)	No	14.09	3.09	5.71	2.52	4.46*
	Yes	15.88	3.53	7.57	6.32	13.51*
Exercise	Yes	14.60	2.84	7.56	4.89	8.72
	No	15.07	4.17	3.85	1.89	5.81

^a^17% missing values, ^b^10% missing values

*p < 0.001 (significant after Bonferroni correction)

In sensitivity analysis, we repeated univariate logistic analysis by treating independent variables of depression, anxiety, and MI as continuous variables, and the results of associations between neonatal outcomes and depression, anxiety, and MI were non-significant (Supplementary Table 1).

After Bonferroni correction, only parity status and SGA were significantly associated (See Table 2). We created a logistic regression model with SGA as the outcome variable and parity status as the single predictor, adjusting for maternal age and partner status. We did this both for complete cases and with multiple imputation. Both types of analysis showed that primiparous women were more than three times as likely (OR: 3.31, 95% CI: 1.66-6.59; OR: 3.27, 95% CI: 1.62-6.62; respectively) to have babies with SGA compared to multigravida (See Table 3).

**Table 3:** Odds ratios (95% CI’s) from logistic regression model of SGA with parity status as independent variable (adjusted for mother’s age and marital status)

Model	Predictor	Type of analysis	Small for gestational age‡ (OR (95% CI))
1	Parity (Primiparous)	Complete cases (n 461)	3.31 (1.66-6.59)*
2		Multiply imputed data (n = 499)	3.27 (1.62-6.62)*

^‡^10% missing values

*p < 0.05

We then examined the possible relationships between neonatal outcomes and MI, depression, and anxiety while treating MI, depression, and anxiety as continuous variables. Similarly, we performed this procedure for complete cases and with multiple imputation. None of the three variables was associated with any adverse neonatal outcome, in either type of analysis (see Supplementary Table 1).

The associations between the other variables (i.e., age, marital status, ethnic background, education level, financial status, physical activities, and alcohol use) and neonatal outcomes were not significant.

## Discussion

To the best of our knowledge, this is the first study to report the relationship between antenatal MI and neonatal outcomes. We did not find any association between neonatal outcomes and MI, antenatal depression, and anxiety. The lack of research on perinatal MI and its effects on the developing fetus limits our ability to discuss the findings in relation to other literature, but the growing research on the relationship of MI with depression, and anxiety serves as a basis for the discussion.

### Shared properties among mood instability, depression, and anxiety

The fact of non-significant relationships among antenatal MI, depression, anxiety, and neonate outcomes may contribute to the argument that depression, anxiety, and MI share some common properties. First, comorbidity. Depression and anxiety comorbid in 63-67% of the patients with major depressive disorder in non-perinatal women [55], while a study of 4,451 postpartum women in the US reported that of the 18% of women who experienced anxiety, 35% also reported postpartum depressive symptoms [56]. The comorbidity between MI and depression was estimated to be 60.9% according to a population study administrated in the UK [57]. Second, neuroticism and negative affect. Studies that examined the positive relationship of MI with depression and anxiety in general and clinical samples have explained by alluding to shared correlates: neuroticism and trait negative affect (NA) [32, 58, 59]. For example, MI is a core feature of neuroticism and also has a strong link with trait NA that are identified as risk factors for both depression and anxiety [27, 60]. Some have suggested that individual differences in neuroticism and NA are central to understanding comorbidity among psychopathologies (e.g., depression, anxiety) [27, 29, 61–63]. Third, stress. The comorbidity between depression and anxiety has been conceptualized in a tripartite model [64]. The model suggests that depression and anxiety have their distinct symptoms, for example, anhedonia for depression and hyperarousal for anxiety, but they share a central common ‘distress’ component. The shared general distress factor is manifested both as a transient state and as a more stable trait [64], and is in line with an internalizing factor in depression, generalized anxiety, and social anxiety [65–67]. Possible bidirectional effects between stress and MI have been proposed. As extreme shifts in mood that last from a few hours to a few days, MI may be a result of interpersonal stress, and high levels of MI may also lead to stressful life events (e.g., the break-up of a relationship, loss of a job) [59]. Fourth, emotion dysregulation. Studies revealed that emotion dysregulation appears to play an important role in anxiety and depressive disorders. For example, emotion dysregulation may create cognitive and functional difficulties in individuals with anxiety and/or depression, such as decreased awareness, poor understanding, inhibited or inappropriate expression, and difficulty managing emotions [68, 69].

### Relation to previous studies

Antenatal depression and anxiety were not associated with adverse neonatal outcomes in the current study that is in agreement with prior studies [16, 18, 20, 70], whereas other studies have reported significant relationships between them [12, 19, 71]. The major reasons for the inconsistent findings might be related to differences in methodology, measurements, sample size or composition, the timing of assessment of depression and anxiety, settings, and variation in accounted for potential confounders [19, 20, 72].

Being primiparous was identified as a significant risk factor for SGA (both in complete-case and multiply imputed data) in the current study, which is consistent with some previous studies [73, 74]. A meta-analysis of 41 studies found that being primiparous increased a woman’s risk of having a baby with LBW and SGA [73]. Parity influences the growth of the placenta and its efficiency, which is related to uterine blood flow, oxygen availability, nutrient exchange, and endocrine regulation of the fetus [74, 75].

### Implications

From a clinical practice perspective, clinicians tend to focus on depression and anxiety. Mood instability is not routinely assessed in a check-up for perinatal women, perhaps due to the perception of perinatal MI as a normal part of a woman’s life, and the propensity for investigating only diagnostic conditions. However, given the evidence of perinatal women experiencing a higher level of MI, a strong link between MI and depression and anxiety in non-perinatal populations [30, 76], and correlation of MI with depression among perinatal women cross-sectionally and prospectively, antenatal MI has the potential to be a risk factor for antenatal depression and anxiety. Therefore, routine screening of MI in clinical and primary healthcare settings may identify women who are at risk for developing depression or anxiety, which could provide another opportunity for prevention, early detection, and early intervention of mood symptoms in perinatal women. As mothers’ mental health has a profound impact on neonatal outcomes and child mental and physical health, more research is required to further understand mood states in pregnancy and neonatal outcomes including longitudinal studies with larger sample sizes.

### Limitations

First, although this is a relatively large sample, the participants were predominantly Caucasian, married, with post-secondary education, and with higher family income, which limits the generalization of the findings. According to the 2016 Census of Canada [77], Indigenous people accounted for 15.6% of the total population of Saskatchewan compared with 7.2% in this sample. Second, our results might not be generalizable to other populations, particularly those with higher rates of preterm birth and malnutrition during pregnancy [78, 79]. Third, for the data analysis, there were cell sizes below 20 for low birth weight x depression and for low birth weight x exercise. The non-significant associations for these variables could be a result of inadequate power due to the low sample size. To better understand the adequacy of the sample, we performed a posthoc power analysis of our logistic regression models and found that the effect sizes of our predictors needed to be 20 percent or greater. MI, depression, and anxiety reached this effect size for at least one adverse outcome, but not for the majority. In adjusting for multiple comparisons, some of our findings were no longer significant, so replication of our analysis with larger samples of mother-infant dyads, and focusing on only a few key factors/determinants, may help to identify whether there are in fact differences/associations. Fourth, the measure of depression, anxiety, MI, and other psychosocial and behaviour variables relied on participants’ subjective report, which could be influenced by women’s current thoughts, feelings, and consideration of social desirability particularly, reporting antenatal smoking, alcohol consumption, and drug use [80, 81]. Fifth, MI holds a temporal property since mood fluctuations can change from moment to moment, which is unlikely to be recalled accurately, such as which day and what time during the day it occurred [82]. Different methods have been used to capture the rapid shift of moods (MI) including Ecological Momentary Assessment and smartphone [83, 84]. Finally, the VAS used to assess antenatal MI is not previously validated. Due to a lack of validated instruments in measuring perinatal MI, studies often utilize non-validated measures [21, 34]. There is a need for validating existing instruments in perinatal women or developing assessment tools that are specifically relevant to perinatal MI.

## Conclusion

The current study expands our knowledge on mood and anxiety symptoms during pregnancy, and their relations to neonatal outcomes. Antenatal MI, depression, and anxiety were not found to be risk factors for adverse neonatal outcomes in a country with well-developed welfare systems. Further studies are needed to explore associations with healthcare accessibility and utilization during pregnancy, and possible long-term effects of maternal MI on children’s development.

## Supplementary Information

**Additional file 1: Supplementary Table 1**. Odds ratios (95% CIs) in models with continuous independent variables.

## Data Availability

The datasets used and analyzed in this study are available from the corresponding author on reasonable request.

## Abbreviations

AGA: Appropriate for gestational age.
CI: Confidence intervals.
EPDS: Edinburgh Postnatal Depression Scale.
FIP: Feelings in Pregnancy and Motherhood Study.
g: Grams.
LBW: Low birth weight.
LGA: Large for gestational age.
MI: Mood instability.
N: Sample size.
OR: Odds ratios.
PTB: Preterm birth.
SD: Standard deviation.
SGA: Small for gestational age.
T1: Early pregnancy:17.4 ± 4.9 weeks.
T2: Late pregnancy:30.6 ± 2.7 weeks.
VAS: Visual Analogue Scale.
