# Supplementary material for: Mood instability, depression, and anxiety in pregnancy and adverse neonatal outcomes
Source: BMC Pregnancy Childbirth. 2021 Aug 25;21:583. doi: 10.1186/s12884-021-04021-y (PMC8385792; doi:10.1186/s12884-021-04021-y)
Supplement: Supplementary file 1 — Additional file 1: Supplementary Table 1. Odds ratios (95% CIs) in models with continuous independent variables. [file 12884_2021_4021_MOESM1_ESM.docx]

| **Supplementary Table 1. Odds ratios (95% CIs) in models with continuous independent variables** | | | | | | |
| --- | --- | --- | --- | --- | --- | --- |
| **Variables** | **Predictor(s)**  **entered** | **Apgar 1 minute score <7 †** | **Apgar 5 minute score <7 †** | **Pre-term birth (< 37 weeks)‡** | **Low birth weight (<2.5 kg) ‡** | **Small for gestational age‡** |
|  |  |  |  |  |  |  |
| Depression | EPDS (univariate) | 1.01 (.95-1.07) | 1.03 (.92-1.15) | 1.03 (.95-1.11) | 1.00 (.90-1.10) | 1.01 (.94-1.08) |
|  | EPDS + maternal age, partnered status | 1.01 (.95-1.07) | 1.03 (.92-1.16) | 1.02 (.95-1.11) | 1.00 (.90-1.10) | 1.00 (.93-1.07) |
| Anxiety | EPDS Anxiety (univariate) | 1.04 (.91-1.19) | 1.06 (.81-1.38) | 1.05 (.88-1.27) | .93 (.74-1.18) | 1.04 (.88-1.23) |
|  | EPDS Anxiety + maternal age, partnered status | 1.05 (.92-1.21) | 1.06 (.81-1.40) | 1.04 (.86-1.26) | .92 (.73-1.16) | 1.02 (.86-1.21) |
| Mood instability | MI (univariate) | 1.01 (.98-1.03) | .99 (.94-1.04) | 1.01 (.98-1.05) | .98 (.94-1.03) | .99 (.95-1.02) |
|  | MI + maternal age, partnered status | 1.01 (.98-1.03) | .99 (.94-1.04) | 1.01 (.98-1.05) | .98 (.94-1.03) | .98 (.95-1.01) |
|  |  |  |  |  |  |  |
